# Effects of standard training in the use of closed-circuit televisions in visually impaired adults: design of a training protocol and a randomized controlled trial

**DOI:** 10.1186/1472-6963-10-62

**Published:** 2010-03-10

**Authors:** Marloes C Burggraaff, Ruth MA van Nispen, Bart JM Melis-Dankers, Ger HMB van Rens

**Affiliations:** 1 VU University Medical Center, Department of Ophthalmology and the Institute for Research in Extramural Medicine (EMGO), PO Box 7075, 1007 MB Amsterdam, The Netherlands; 2 Royal Visio, National Foundation for the Visually Impaired and Blind, PO Box 144, 9750 AC Haren, The Netherlands; 3 Elkerliek Hospital, Department of Ophthalmology, PO Box 98, 5700 AB Helmond, The Netherlands

## Abstract

**Background:**

Reading problems are frequently reported by visually impaired persons. A closed-circuit television (CCTV) can be helpful to maintain reading ability, however, it is difficult to learn how to use this device. In the Netherlands, an evidence-based rehabilitation program in the use of CCTVs was lacking. Therefore, a standard training protocol needed to be developed and tested in a randomized controlled trial (RCT) to provide an evidence-based training program in the use of this device.

**Methods/Design:**

To develop a standard training program, information was collected by studying literature, observing training in the use of CCTVs, discussing the content of the training program with professionals and organizing focus and discussion groups. The effectiveness of the program was evaluated in an RCT, to obtain an evidence-based training program. Dutch patients (n = 122) were randomized into a treatment group: normal instructions from the supplier combined with training in the use of CCTVs, or into a control group: instructions from the supplier only. The effect of the training program was evaluated in terms of: change in reading ability (reading speed and reading comprehension), patients' skills to operate the CCTV, perceived (vision-related) quality of life and tasks performed in daily living.

**Discussion:**

The development of the CCTV training protocol and the design of the RCT in the present study may serve as an example to obtain an evidence-based training program. The training program was adjusted to the needs and learning abilities of individual patients, however, for scientific reasons it might have been preferable to standardize the protocol further, in order to gain more comparable results.

**Trial registration:**

http://www.trialregister.nl, identifier: NTR1031

## Background

Vision loss is an increasing medical and social problem. In Western countries, vision loss is mainly age-related[1]. Currently there is no cure for most of the age-related eye disorders, leaving rehabilitation as the only option. Rehabilitation programs have the potential to restore independence and improve the quality of life of visually impaired persons[2].

In the Netherlands, two main types of low vision rehabilitation exist: mono-disciplinary optometric services and multidisciplinary rehabilitation centers (MRCs). Optometrists have had special training in prescribing low vision aids (LVAs), adjusting them to the patients' remaining visual functions and offering instructions in the use of the aids. MRCs offer training in activities of daily living, advice on adaptations in the home environment and individual or group counseling, in addition to advice about LVAs[3].

In both services, reading problems are most frequently reported[4], some of which can partly be solved by prescribing LVAs. Use of LVAs can reduce reading problems as well as other nearby visual tasks, helping to diminish or slow down the disability process and helping to maintain independence. A closed-circuit television (CCTV) is a type of LVA that is prescribed to patients with moderate to severe vision loss[5], because it enables higher magnifications than optical magnification aids[6]. CCTVs have many other advantages over optical magnification aids such as: contrast enhancement, image manipulation, reduction of aberrations, less critical focus, more natural working distances, better posture, binocularity and a longer duration of reading [6-8]. However, CCTVs also have some disadvantages. For instance, they are expensive (costing around $2,500), most types are not portable and it takes effort to learn how to use a CCTV effectively. It is well known among low vision professionals that many CCTVs are rarely used; some are even returned to the provider because patients find it hard to use these devices. Watson et al.[9,10] reported that 15% of all prescribed LVAs were abandoned after 12-24 months. However, most patients continued using their CCTV, which was also found by Goodrich et al.[11]. On the other hand, only 87% demonstrated effective use [11]. Reasons for abandonment of assistive devices are failure to improve function and quality of life [12], continued use of LVAs is positively correlated to having a helper in the home [9,10].

Training has proven to be effective in learning how to use CCTVs[6,11,13-20]. However, published information on an optimal training program is not available[21]. Although some exercise books on training in the use of CCTVs exist within the Dutch MRCs, they are rarely used in daily practice and standardized protocols are lacking. Furthermore, most studies on CCTV performance have been carried out in the U.S. in inpatient centers [6,11,13-17,19], whilst in the Netherlands, as well as in many other countries, most MRCs offer outpatient rehabilitation services. Therefore, the first aim of the study was to develop a standard program, adjusted to the Dutch situation, to train individuals with visual impairment how to use CCTVs. However, when developing an evidence-based rehabilitation program, it is necessary to evaluate this new standard training with respect to effectiveness in a randomized controlled trial (RCT). To our knowledge, only two RCTs have been conducted to evaluate reading performance with CCTVs[20,21]. Moreover, these RCTs present contradictive results. Faubert and Overbury[20], for example, found that basic use of CCTVs, without specific training, did improve reading speed. In contrast, Peterson et al.[21] reported that familiarity with a CCTV had no significant effect on reading speed or task performance. These trials had substantial limitations, the first trial concentrated on young healthy subjects[20], and the other concentrated on visually impaired patients who received only two minutes of training in the use of a CCTV. Furthermore, the latter study compared reading performance with a CCTV to reading performance with the patient's own optical magnifier[21]. Therefore, the present study will disclose if training in the use of CCTVs is effective by comparing a treatment group (patients who receive training) versus a control group (patients who will not receive these instructions).

In the current paper, the development and final content of the standard protocol for training in the use of CCTVs are described, as well as the design and methodology of the RCT that will evaluate the effectiveness of this training. If the standard training is effective, the protocol will be the new Dutch national instruction for CCTV trainers.

## Methods/Design

### Development of a new standard training protocol in the use of CCTVs

#### Evidence from previous studies

The first step in developing a new standard protocol for training in the use of CCTVs in the Netherlands, was to search the literature for training methods, durations and frequencies. Two reviews on vision enhancement devices, summarized the outcomes and contents of training programs of studies that concentrated on CCTVs[22,23], these and other studies were investigated more intensively. The quantitative focus was on the amount, duration and frequency of training in the use of CCTVs. The amount of training with a stand mounted CCTV differed in the various studies from five [17] to at least fifteen training sessions[6,9,10,13]. Research by Goodrich et al. [15-17] showed that reading performance did not significantly increase with additional training after five sessions. The durations of individual sessions was typically 40-60 minutes [15-18]. In contrast, in the study of Peterson et al.[21] patients received two minutes of active training after the device was explained and demonstrated. Training was given on consecutive days[14,17] or once or twice a week[18]. The qualitative focus was on the content of training, which was similar in the various studies. All programs focused to some extent on ergonomics, operating the device, tracking, skimming, reading and writing. One program also included trouble-shooting the device[6]. During training sessions different reading and writing materials were used. In all studies patients actively used a CCTV and performed practical tasks. Faubert and Overbury[20], and Lund and Watson[24] both reported the importance of arm movements and active training strategies.

#### Reaching consensus on the content of the CCTV training protocol

The second step in developing the protocol was to obtain information about current training methods from the participating MRCs, to be able to design a training protocol that would reflect the daily practice of the MRCs and that would be easy to implement after the study has been finished. The MRCs in the Netherlands are operated by three main organizations for low vision care: Visio and Sensis (as of January 2010: Royal Visio), and Bartiméus. Patients were included at nine regional centers: Bartiméus participated with one MRC in the east of the Netherlands, Sensis participated with three MRCs in the south of the Netherlands and Visio participated with five MRCs in the north and southwest of the Netherlands. Of each organization one representative (a clinical physicist), with knowledge of CCTVs and training in their use, discussed with the low vision therapists (mostly occupational therapists) the duration, frequency and content of training programs given prior to the start of the present study. Furthermore, they discussed possible improvements, which could be incorporated into the new training protocol. A focus group was then organized, attended by the representatives of the three organizations and the authors, in which the outcome of the literature search and the outcomes of the discussions within each organization were further explored. It came to light that a considerable number of low vision therapists did not offer structured training. The duration and amount of training, as well as the manner in which patients were trained, differed per trainer and between the different MRCs. Some exercise books on training in the use of CCTVs existed within the MRCs, which could be used in the design of the protocol. Within the focus group an initial design for the protocol was constructed, which was then adapted by the authors. A discussion group was held in which the adapted version of the protocol was discussed with 12 low vision therapists, and the protocol was sent, twice, to 36 therapists at the nine participating MRCs. Only minor revisions were necessary, before consensus regarding the final content of the protocol was reached.

#### Final content of the CCTV training protocol

In designing the final content of the training protocol, the age of the actual users of CCTVs was taken into account. In the Netherlands the majority of CCTV users is estimated to be above the age of 70 years. Many older visually impaired patients cannot concentrate for long periods. Therefore, based on the experience of the low vision therapists, two 30 minute sessions separated by a break of 15 to 30 minutes were scheduled. The frequency of training was once a week. The amount of training depended on the learning strategies of the individual patients. Patients were trained until they had practiced with every assignment or until no further improvement could be reached.

Table 1 summarizes the main components of the CCTV training protocol. The protocol focused on various aspects, similar to those in previous studies such as: ergonomics, basic instructions for operating the CCTV and tracking skills with the X-Y table [6,15-19,24]. When participants were familiar with the basic aspects of the CCTV, reading was practiced with different reading materials. Furthermore, looking at pictures and photographs was practiced, since this demands some special skills (e.g. with respect to preventing glare). Next, participants practiced writing with their CCTV, using different exercises. Finally, participants were asked which hobbies they would like to practice and some examples were given. Throughout the training program, participants practiced with the easier assignments before they tried the more difficult ones (easy-to-difficult strategy[17]). Hands on training was given [17], with direct feedback to the participants, to avoid the adoption of incorrect habits. Training was given by low vision therapists from the three organizations, who scored the patient's progression by registering all the assignments patients could carry out and also which particular assignments patients found difficult.

**Table 1:** Main components of the standard training protocol in the use of CCTVs.

Components	Details	
Elements - Ergonomics	- Position of CCTV in the room - Posture for working with CCTV - Height of CCTV and working distance	
Elements - Basic operation instructions	- On/off switch - Magnification - Image contrast - Position of lights and camera - X-Y table and basic tracking skills	
Elements - Reading (different materials)	- Basic reading assignments (e.g. reading words/small sentences) - Newspapers, books, magazines, postcards, medicine bottles etc.	
Elements - Watching pictures	- Pictures and photographs with different pixel amounts	
Elements - Writing (different materials)	- Basic writing assignments (e.g. drawing crosses in boxes) - Virgin and zoned paper, cheques, forms	
Elements - Hobbies and other CCTV skills	- Interests and hobbies of the patients are discussed and practiced (e.g. painting, fiddling, sewing, drawing etc.)	
Applied techniques	- Hands on training - Easy-to-difficult strategy	
Number of sessions	- Variable, adjusted to the individual patients need	
Frequency	- Once a week	
Duration	- Sixty minutes per session	
Format	- Face-to-face	
Location	- Patients' home environment	

### Design of the randomized controlled trial

The study is a multi-center masked RCT conducted at nine Dutch regional MRCs to evaluate the effectiveness of a new standard protocol to train low vision patients how to use a CCTV. Table 2 lists the inclusion and exclusion criteria.

**Table 2:** Inclusion and exclusion criteria.

Inclusion criteria	Exclusion criteria
Visually impaired with an indication for a (stand mounted) CCTV	Cognitive deficits
Age ≥ 18 years	
Able to speak and understand Dutch	
Acceptance of the conditions of the study	

#### Sample size and treatment effect

Although other outcome measures were also used, power calculations were based on reading speed, which has been the primary outcome of previous studies on training in the use of CCTVs[6,11,13-21]. Subjects in the study of Goodrich et al.[16], were comparable to subjects in the present study and, to our knowledge, this was the only study that provided a mean reading speed in words per minute including standard deviations at the beginning and end of training in the use of CCTVs. Therefore, power calculations were based on data these authors presented. In their study, mean reading speed with a CCTV was 71.8 words per minute (SD 33.5) at the end of the first training session and 89.2 words per minute (SD 37.9) at the end of the last (15^th^) training session. Sixty-two participants in each treatment arm of the trial provide a power of 0.85 with alpha 0.05, to detect differences between the training and the control group of 20 wpm, after accounting for 17% of participants who may miss the three month evaluation[25].

#### Randomization

There were different ways in which patients entered the MRCs, e.g. through referral by an ophthalmologist, a general practitioner or an optometrist, through advice from friends and relatives, or through advertisements in papers or on the Internet. Low vision specialists at the MRCs examined the visual function of both eyes of all patients (e.g. visual acuity, visual field, contrast sensitivity and reading speed) and explored their rehabilitation needs. This was followed by the low vision device evaluation, during which patients tried several LVAs. CCTVs are typically prescribed to patients with very low visual acuities, i.e. <0.05 Snellen[5]. Near addition and prism spectacles were provided for the working distance if necessary. In the Netherlands, the cost of LVAs and training in their use are reimbursed. If a CCTV was indicated, patients were screened for eligibility. Eligible patients received information about the study and a baseline questionnaire. Patients who administered the questionnaire and signed informed consent were included in the study. Participants were then randomized to either the treatment group or to the control group. Randomization was performed by research personnel, not involved in the study, using a computer-generated allocation scheme based on blocks of two, stratified by the nine sites. The random assignments to the treatment arms were sent by email to the MRCs.

Participants in the treatment group received training in the use of their CCTV according to the new standard protocol, from the low vision therapists of the MRCs. Participants in the control group did not receive training in the use of their CCTV. However, all participants (in both groups) received the usual instructions from the various suppliers when the CCTV was delivered. Suppliers were unaware of the present RCT, which means that their instructions were not bounded by a predefined protocol. Therefore, the instructions may differ between participants from none (delivery of the CCTV without instructions) to basic instructions for operating the CCTV (similar to the chapter in the CCTV training protocol). To compensate for lack of treatment in the control group, training was offered after their follow-up measurements had been performed.

Participants and trainers were aware of the random assignment, however, the main investigators who rated the CCTV performance and the treatment effect were masked to the treatment allocation until after the final analyses of the primary and secondary outcomes. The study protocol was approved by the Medical Ethics Committee of the VU University Medical Center, Amsterdam, and conducted according to the principles of the Declaration of Helsinki. All participants gave written or verbal informed consent.

#### Outcome measures

The effects of training were evaluated in terms of change in visual reading ability (primary outcomes) and perceived quality of life and related topics (secondary outcomes). Table 3 lists the assessments and data collection performed at each time point for patients in both treatment arms.

**Table 3:** Assessments.

Type		Screening	Baseline	Pre-training	Post-training (three months follow-up)
Low Vision Examination		X			
Low Vision Device Evaluation		X			
Eligibility screening		X			
Demographics			X		X
Euroqol 5 Dimensions [34]			X		X
Euroqol Thermometer [34]			X		X
LVQOL [35]			X		X
CES-D [36]			X		X
AVL [37]			X		X
Radner Reading Chart [28]				X	X
Test Technical Reading 345678 [31]				X	X
Reading comprehension test [32]				X	X
Dutch version of the Activity Inventory [33]				X	X
Use and satisfaction Questionnaire				X	X

Change in the primary outcomes of visual reading ability was measured using several tests, to obtain: reading speed, reading comprehension and patients' skills to operate the CCTV (e.g. focusing, setting contrast, adjusting magnification, positioning and moving the material on the X-Y table[11]). The primary outcome measure was reading speed, using the Radner Reading Charts (RRCs)[26,27], of which recently a Dutch version was developed[28]. The RRCs have sentences that are highly comparable in terms of lexical difficulty, syntactical complexity, word length, number and position of words. The charts consist of 14 sentences of which the print size graduates with 0.1 log unit steps. The Dutch RRCs showed, even as the original German RRCs to have a high inter-chart and test-retest reliability in subjects with normal and impaired vision due to maculopathy[29,30]. Unpublished data by Burggraaff et al. confirmed the high inter-chart and test-retest reliability of the Dutch RRCs, in a heterogeneous population with different causes of visual impairment. Therefore, the RRCs are considered to be the best available method for assessing reading speed and reading acuity in the Netherlands.

Reading comprehension is another visual reading ability outcome measure that was used. Participants using a CCTV may have difficulty understanding their reading materials, since magnification reduces the amount of text displayed at once. In the Netherlands, there are no validated tests available for measuring reading comprehension in adults or in low vision patients. Therefore, two tests used in pre-school were included after consulting three academics involved in developing Dutch reading comprehension tests. The first test measures technical reading, which is considered to precede reading comprehension, and is called 'Test Technical Reading 345678'[31]. The test consists of 140 words with ascending word length. In 90 seconds patients read as many words as possible. The second test consisted of two texts (level of 8^th ^grade school children) with quiz questions to assess reading comprehension [32]. For every test, including the RRCs, different versions were used during the two home visits to avoid learning effects. It is expected that reading comprehension is related to reading speed, but also to education level and other variables. This will be taken into account in the analyses.

To estimate the treatment effect of tasks performed in daily life, a relevant part of the Dutch version (unpublished data by Bruijning et al.) of the 'Activity Inventory' was used[33]. Questions about reading and near tasks were selected and administered in a structured interview during the home visits. Answers were entered directly into a laptop. The diversity of tasks performed with the CCTVs (e.g. reading newspapers and books, writing, puzzling, sewing) as well as the duration and frequency the CCTVs were used, was obtained with a CCTV diary. The participants were requested to register their daily use of the CCTV (e.g. 10.00-10.30 a.m. reading the newspaper).

To assess the secondary outcome measures, i.e. changes in quality of life and related topics, the same questionnaire distributed at baseline, was distributed among the participants once more during the 3-month evaluation point. The questionnaire consisted of validated questionnaires to explore demographics and health status (EuroQol 5 Dimensions and EuroQol Visual Analogue Scale[34]) and other relevant measures of outcome such as vision-related quality of life (Low Vision Quality Of Life questionnaire (LVQOL)[35]), depression (Center for Epidemiological Studies Depression scale (CES-D)[36]) and adaptation to vision loss (Adaptation to age-related Vision Loss scale (AVL)[37]), as these latter two might influence ability or motivation to use the CCTV.

Immediately after patients had received their CCTV (before the start of the training program in the treatment group) baseline measurements (i.e. the visual reading ability outcomes and the 'Activity Inventory') were administered by students during a home visit. The delivery of CCTVs, took approximately six weeks (range 3-231 days, see Table 4), therefore, the final outcome measurements were taken four to six months after baseline. A home visit was considered the best way to observe patients using their CCTV in their own environment. Five students, from Social and Health Sciences, have performed all the home visits. They received special instructions in administering the tests. To enable objective measurements, videotapes were used to register the time spent reading, to record reading mistakes whilst participants were reading aloud and to score the patients' skills to operate the CCTV. The tapes are currently rated by two independent investigators unaware of the treatment allocation using a standard rating protocol.

**Table 4:** Participant characteristics.

Characteristics		Mean (SD)	Median [IQR]	Percentage	Range
Age (years)		78 (12)	80 [72-86]		34-95
Gender (female)				60%	
Education (years)		9.8 (2.8)	10 [9-11]		5-16
Co-morbidity (yes)				72%	
Living situation (independent)				86%	
Social status (married)				51%	
Referred by an ophthalmologist				37%	
Previous use of an LVA, other then a CCTV				94%	
Previous contact with an MRC				32%	
Patients invited by Visio				41%	
Patients invited by Bartiméus				38%	
Patients invited by Sensis				21%	
Time between visit MRC and delivery CCTV (days)		42 (32)	34 [17-64]		3-160
Time between delivery CCTV		13 (6.5)	12.5 [8-16]		3-31
Time between pre and post-training measurements		96 (8.8)	94 [91-99]		81-134

Participants in the control group received only the instructions as provided by the supplier. All participants (in both the treatment and control group) were asked to report the specific contents of these instructions at the first home visit.

#### Statistical analysis

The analysis will be based on the intention-to-treat principle. To test for differences between groups univariate techniques and mixed regression analyses will be used with respect to the primary and secondary outcome measures at the follow-up assessments. Models will be adjusted for relevant confounders, including age, visual acuity and possible baseline differences. Data will be analyzed using the software package SPSS 15.0 for Windows.

#### Participant characteristics

**Figure 1:**
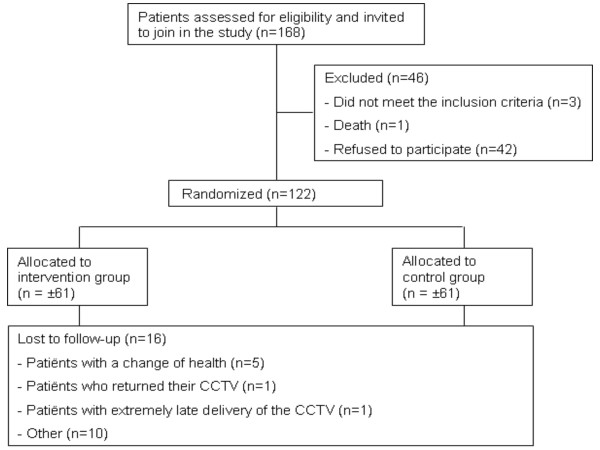
**Overview of the design of the RCT**.

Patient recruitment started in April 2008. As of 21 August 2009, 168 patients have been invited to participate in the study and have been screened for eligibility. Four patients were excluded, three patients were ineligible and one patient died before consenting. Forty-two patients did not want to participate in the study, 122 patients responded and participated in the study (73%). Table 4 lists the characteristics of the participants and Figure 1 shows an overview of the design of the trial.

## Discussion

In the present study consensus was reached by low vision therapists, clinical physicists and researchers in the field of low vision on a standard protocol for training in the use of CCTVs. The protocol consists of various chapters including several exercises within each chapter. The focus of the training protocol is on ergonomics, basic operation skills, reading, writing, looking at pictures and photographs, and carrying out hobbies. An RCT was conducted to test the effectiveness of the protocol by comparing an intervention group, i.e. patients who receive both instructions provided by the supplier and the new standard training, with a control group, i.e. patients who receive only the instructions from the supplier.

The study will concentrate on predictors of training effects. For example, there might be a 'dose-response' relation between the number of sessions or the time per session and the effect of treatment in terms of primary [15-17] and secondary outcomes. Moreover, we expect that low vision specialists will be able to use our results in deciding which patients will be eligible for training in the future, for example patients with a certain visual acuity or patients who suffer from non-visual co-morbid conditions. Finally, optometrists may have a better indication for referring patients to MRCs for additional training. Moreover, by studying patient files and obtaining data from the participants during the home visits, this study will provide knowledge about the process of counseling and prescribing CCTVs to visually impaired adults (e.g. knowledge about which patients were prescribed a CCTV and about their rehabilitation needs). Consequently, the process of delivering CCTVs will become more transparent. At the time the study was conducted, the exact content of the instructions suppliers provide when they deliver CCTVs to patients' homes was unknown, as well as the time taken for these instructions. We decided not to inform suppliers about the study, to avoid them starting to over perform on their usual instructions, which may vary somewhere between no instructions to basic instructions on how to operate the CCTV. If they would have been informed, a smaller effect of the new standard protocol for CCTV training may be expected. Also, we did not know in advance which supplier would be involved in delivering a CCTV to a particular eligible patient. This depends on the insurance company of the patient and the rehabilitation center that has CCTVs of specific providers on display. In the course of the present study information about these instructions was obtained from participants. Comparability between the treatment and control group with regard to the instructions of the suppliers will be investigated.

There are some limitations to the present study. First, the recruitment of participants by the MRCs took longer than expected. Reasons for this delay were: lack of eligible patients, lack of interest of clients, workload of low vision specialists who were required to invite eligible participants and ethical considerations of a low vision specialist who had conscientious objections to inviting participants with a need for CCTV training. This low vision specialist only invited patients who needed a CCTV, but who could manage without training, so that it would not matter if a patient would be randomized into the control group. Although several actions had been undertaken to convince the low vision specialist of the purpose of the study, and that all eligible patients with a CCTV indication should be invited, it did not work out properly. For this particular MRC, this has caused a selection bias which may lead to an underestimation of the treatment effect which influences generalizability of the results. Therefore, data will be analyzed with and without patients from this specific site.

Second, we only focused on CCTVs with stand mounted cameras and/or displays, since these are the types that are most commonly prescribed in the Netherlands. In addition to stand mounted CCTVs, there are 'mouse' style CCTVs and CCTVs with handheld or head-mounted cameras. All of these devices are more portable than stand mounted CCTVs. However, battery power options tend to be heavy and expensive and, mouse style CCTVs especially, have a limited range of magnification[22]. Head-mounted CCTVs can be used for a wide variety of tasks, nevertheless, patients with macular disease showed better functioning using optical aids compared to head-mounted CCTVs for the majority of tasks in research by Culham et al.[38].

Third, for scientific reasons it would have been preferable to standardize the training protocol further. For instance, to standardize the frequency and the total minutes of training each participant receives, as well as the amount of time practicing with each assignment. However, in daily practice it is very important to amplify care to the characteristics of the individual patient (e.g. learning abilities, endurance and limitations due to co-morbidity). For this reason, training was adjusted to the rehabilitation needs of the participants as well as to their learning style and learning rate, e.g. when participants had major problems with reading, the reading assignments were practiced more intensely. Another advantage of this method is that when the training program has been proven effective, only a few adjustments might be necessary before implementing the protocol in daily practice.

Finally, it would have been preferable to offer placebo-training to the control group, to rule out a Hawthorne effect. An attention deficit in this group may result in a poorer outcome, e.g. a lower experienced quality of life. However, the study of Reeves et al. did not show significant differences in task performance and quality of life between patients who received conventional low vision rehabilitation enhanced by home visits from a rehabilitation officer (who gave advice and demonstrations on the use of LVAs, supplied alternative LVAs and provided patient support) or from a community care worker (who did service as a control for the contact time with patients)[39].

## Conclusion

We believe that the limitations of the study can be considered small compared to the information the study will provide: a new standard training protocol was developed to instruct visually impaired adults in the use of CCTVs. In addition, the effectiveness will be tested in an RCT, which allows us to provide an evidence-based training.

## Abbreviations

AVL: Adaptation to age-related Vision Loss scale; CES-D: Center for Epidemiological Studies Depression scale; CCTV: Closed-Circuit Televisions; LVA: Low Vision Aid; LVQOL: Low Vision Quality Of Life questionnaire; MRC: Multidisciplinary Rehabilitation Center; RCT: Randomized Controlled Trial.

## Competing interests

The authors declare that they have no competing interests.

## Authors' contributions

MB, RvN, BMD and GvR participated in the conception and design of the study. MB drafted the manuscript, which was revised by the other authors. All authors read and approved the final manuscript.

## Pre-publication history

The pre-publication history for this paper can be accessed here:

http://www.biomedcentral.com/1472-6963/10/62/prepub
